# Kaminari Kagura: the “lightning bolt shape” technique for posterior vitreous detachment induction

**DOI:** 10.1007/s10792-023-02867-z

**Published:** 2023-09-20

**Authors:** Tomaso Caporossi, Gloria Gambini, Antonio Baldascino, Alessandra Scampoli, Matteo Mario Carlà, Stanislao Rizzo

**Affiliations:** 1 Catholic University “Sacro Cuore”, Rome, Italy; 2 Vitreoretinal Surgery Unit, Fatebenefratelli Isola Tiberina Gemelli Isola Hospital, Largo A. Gemelli 8, Rome, Italy; 3 Ophthalmology Unit, Fondazione Policlinico Universitario A. Gemelli IRCCS”, Rome, Italy; 4 Consiglio Nazionale Delle Ricerche, Istituto di Neuroscienze, Pisa, Italy

**Keywords:** Vitreous detachment, Posterior hyaloid, Vitreoretinal surgery, Retina, Vitrectomy, Vitrectome

## Abstract

**Purpose:**

To describe and evaluate the effectiveness of the Kaminari Kagura technique as a posterior hyaloid detachment treatment.

**Study design:**

This was a prospective, consecutive, randomized interventional study.

**Methods:**

This study examined 30 eyes from 30 patients divided into two groups: (1) a Kaminari Kagura group (15 eyes) and (2) a control group (15 eyes) scheduled for vitrectomy with an optical coherence tomography (OCT)-based diagnosis of adherent posterior hyaloid.

**Results:**

The mean time for posterior vitreous detachment (PVD) induction in the Kaminari Kagura group was 58 ± 6.6 s, and that in the control group was 69 ± 9 s (*p* < 0.005). No intra- or post-operative complications were reported.

**Conclusions:**

The Kaminari Kagura technique results in effective posterior hyaloid detachment in less time than that required for posterior vitreous cortex engagement.

**Supplementary Information:**

The online version contains supplementary material available at 10.1007/s10792-023-02867-z.

## Introduction

The induction of posterior vitreous detachment (PVD) is a fundamental step in most vitreoretinal surgeries. Although in most of the cases, it can be easily performed, there are cases in which the hyaloid is strongly adherent due to the young age of the patients, and simple aspiration without a support by a particular method can cause a waste of time. To reduce the time of surgery and the variability of the cases, a standardized, repeatable, and easily applicable approach is needed. Various surgical techniques have already been described. Some authors described the approaches starting from the Weiss ring separation [1, 2]. Other authors suggest techniques starting with the creation of a hole in the posterior hyaloid surface to allow the vitreous to pass through it and help with the separation process.

The induction of PVD to facilitate the passage of fluids into the sub-hyaloid space is done using different instruments, such as intravitreal diathermy [3], a nylon-tipped adjustable brush [4], a short-tipped pick [1], or a microvitreoretinal (MVR) blade [5]. However, the need for creating a hole in the hyaloid with these tools leads to the possibility of complications, such as retinotomies or haemorrhages. Therefore, the Kaminari Kagura technique is proposed as an easy and repeatable way to enable gentle dissection of the posterior hyaloid with just active aspiration without the need for other tools.

## Methods

This prospective, interventional case-series study examined consecutive patients scheduled for pars plana vitrectomy (PPV) at the Department of Ophthalmology, Catholic University of Sacred-Heart Foundation *Policlinico Universitario A. Gemelli*, Rome, Italy. The study included 30 eyes of 30 patients, of which 15 eyes were in the Kaminari Kagura group, and 15 were in the control group. All patients underwent a complete ophthalmic examination. In addition, a structural optical coherence tomography (OCT) (Mirante SLO/OCT, Nidek Co., Maehama, Hiroishi-cho, Gamagori, Aichi, Japan) was performed preoperatively on all patients. All patients underwent a retina map scan with an area of 12 mm × 9 mm area, which allows for wide-area diagnosis, including the macula and optic disc, and helps with assessing the status of the PVD.

All the patients had no PVD, according to OCT, were affected by a macular hole (MH) and were scheduled for PPV. The included types of PDV were absent PVD (stage 0), paramacular PVD (stage 1), and vitreoretinal separation from part of the fovea with persistent attachment to the foveola (stage 2). Candidates were excluded if they were beyond stage 3, had entire macular detachment with continuing to the optic nerve, or had complete PVD [6]. Randomization of the two groups was performed preoperatively.

All clinical procedures were conducted according to the principles of the Declaration of Helsinki. The protocol used was approved by the local ethics review board. Written informed consent was obtained from all patients prior to the procedure. The surgeries in the Kaminari Kagura group were performed by T.C., and the surgeries in the control group were performed by two expert surgeons (S.R. and A.B.). Statistical analysis was performed using STATA software version 15.1 (StataCorp. College Station, TX). An unpaired t-test was used to compare the mean values. The significance level was set at *p* < 0.05.

### Surgical technique

**Fig. 1:**
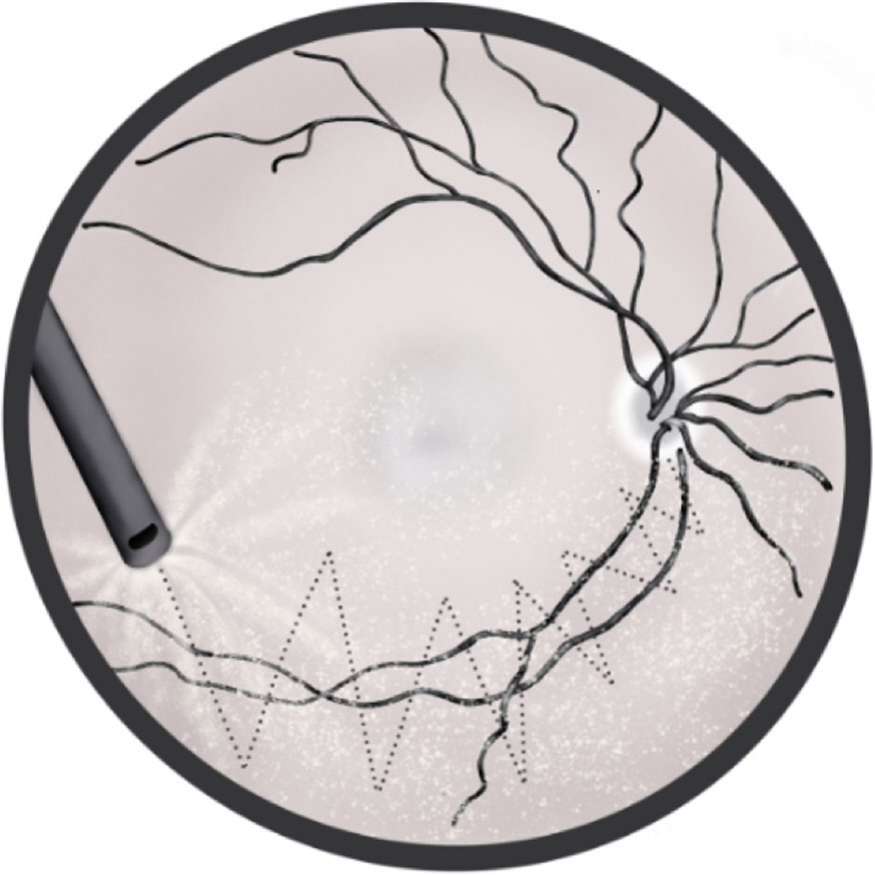
Design of the movement of the vitrectome probe during the induction of the posterior vitreous detachment. The shape of the movement is like to design a lightning bolt on the surface of the retina (Kaminari in Japanese)

**Fig. 2:**
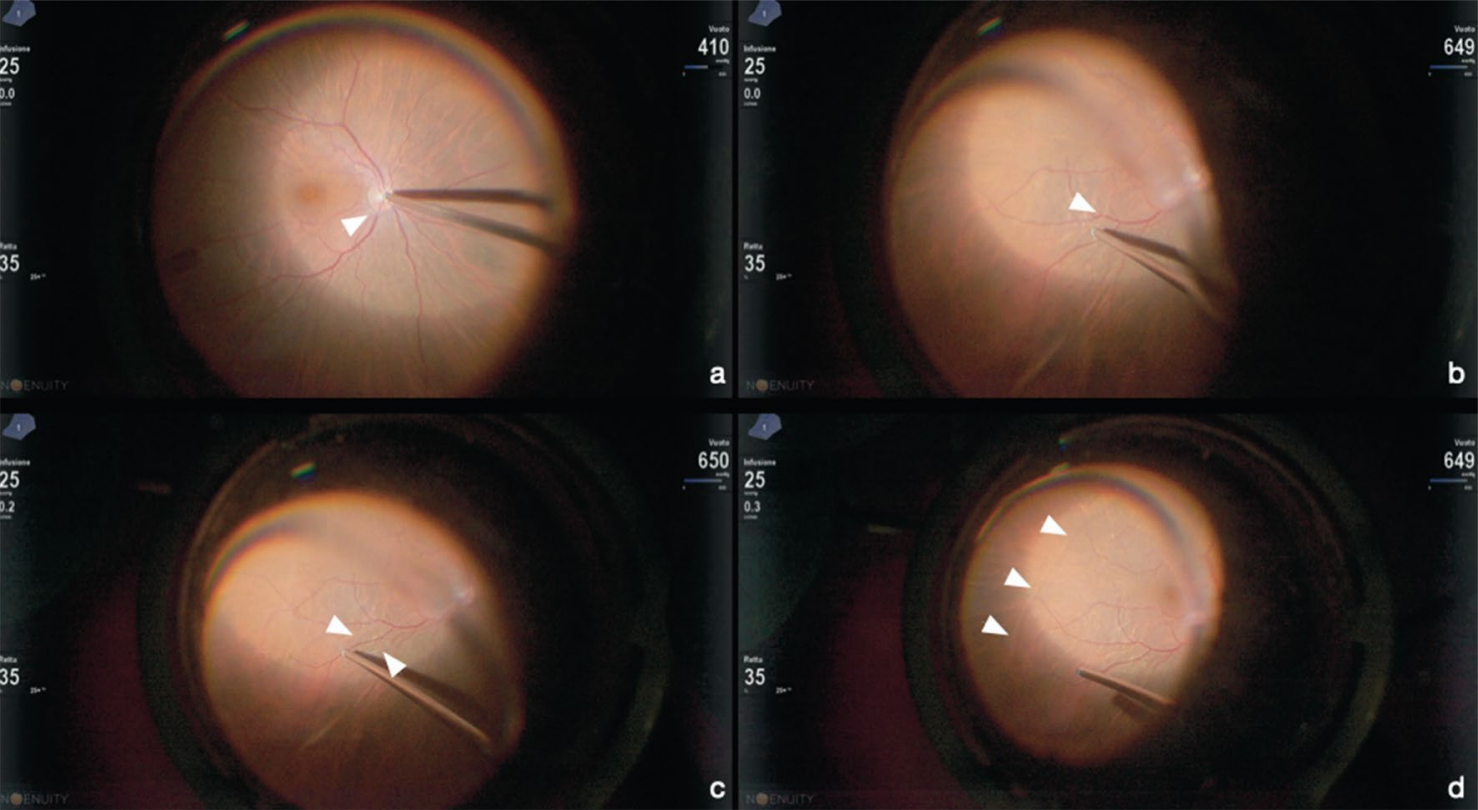
Engaging the posterior hyaloid on the optic nerve head. The white reflex is the sign of the engaged vitreous cortex (white arrow) (**a**). During the movement of the engaged posterior hyaloid reflex (white arrow) (**b**). Advance stage of posterior vitreous engagement (white arrows) (**c**). Posterior hyaloid detachment (white arrows) (**d**)

A 25-gauge PPV was performed in all cases. In the Kaminari Kagura group, after a core vitrectomy, the posterior hyaloid was engaged with a vitrectome probe at the site of the optic nerve head. The vitrectome was moved by following one of the vascular arcades in a zig-zag fashion (lightning bolt shape) (Figs. 1, 2). When the vitreous was sufficiently engaged in the mouth of the vitrectome probe, a vertical movement was used to complete the detachment of the vitreous posterior hyaloid (see supplemental digital content 1)**.**

In the control group, after a core vitrectomy, the posterior hyaloid was gently engaged with the vitrectomy probe at the papillo-macular site and detached in a tangential fashion. The procedure was repeated until the PVD induction. The time to complete the PVD was measured by analysing the intraoperative video recorded by four independent and masked expert operators (G.G., M.M.C., A.S., and G.G.). The times required were recorded for each eye, and the mean value was calculated. The agreement between the time graders was estimated with a Bland–Altman plot. The intraoperative complications were recorded.

## Results

The study included 30 eyes of 30 patients with no PDV according to OCT. All eyes were affected by MH and were scheduled for PPV. The mean time for PVD induction in the Kaminari Kagura group was 58 ± 6.6 s, and that in the control group was 69 ± 9 s (*p* < 0.005). The main difference was in the number times that the procedure was repeated due to the loss of the coupling between the probe and the posterior hyaloid (3 times in the Kaminari Kagura group (20%) and 10 times in the control group (66.6%); *p* = 0.001). During the 3-month follow-up period, no patients had intraoperative or postoperative complications, such as retinal tears, bleeding, or retinal detachment.

## Discussion

The creation of a PVD is critical in PPV for most macular vitreoretinal interface disorders and retinal detachments. However, PVD induction can be a challenging surgical step in young patients and in older patients with a particularly adherent posterior hyaloid or abnormal vitreoretinal interface. Failure to successfully induce PVD can result in poor surgical outcomes, such as persistent MHs and proliferative vitreoretinopathy.

As highlighted by Takeuchi et al. [7], the power of hooking and holding the hyaloid using only active aspiration positively correlates with the dimension of the aperture of the vitrectome probe. When using 25-gauge and 27-gauge, the only way to keep the hyaloid hooked during aspiration is by finding a plane of movement that is parallel to the hyaloid plane and rise very slowly with the probe gently accompanying the detachment of the hyaloid. Otherwise, a sharp vertical movement not accompanied by horizontal movements will cause the loss of the coupling between the probe and the hyaloid.

Otani et al. [8] and Takeuchi et al. [7] both described a method of creating a hole in the posterior precortical vitreous pocket (PPVP). One method used a silicon-tipped needle with aspiration, and the other used a diamond-dusted membrane scraper. They observed retinal haemorrhages, focal whitening spots, and tears. Other groups have described techniques for dissection of the posterior vitreous cortex from the retina starting at the edge of the optic nerve using a pick, a micro-vitreoretinal blade, or a micropick combined with active aspiration [1, 2]. The latter technique is a combination of aspiration and sharp dissection that allows a controlled traction on the vitreous cortex and entry into the sub-hyaloid space with the sharp instruments, which decreases the possibility of retinal trauma.

Although different methods have been described, most surgeons tend to use active suction of the vitreous to cause the PVD, making different attempts around the optic nerve and switching to other methods when this method is insufficient. The PVD induction depends on the capability to engage the vitrectome probe with the posterior hyaloid and is directly proportional to the flow rate of the vitrectomy machine. The evolution of probe design continues to expand the variety of surgical parameters governing the flow rate. Design improvements such as reduced outer probe diameter have led to 23- and 25-gauge transconjunctival sutureless vitrectomy instruments that reduce sclerotomy inflammation [9, 10]. Although smaller-diameter instruments have improved postoperative patient comfort, surgeons have experienced reduced flow rates during vitrectomy [11].

Poiseuille's law can be used to describe the flow rate through a vitrectomy probe, and the variable R represents the thinner lumen radius. However, Poiseuille's equation is valid for laminar flow of an incompressible fluid, and the heterogeneous composition of vitreous complicates the fluid dynamics during vitrectomy. Nevertheless, we can hypothesize that engaging the posterior hyaloid with the vitreous probe is strongly related to the vitrectome diameter, which influences the flow rate. This is why increasing the coupling between the hyaloid and vitrectome with a movement of the probe could result in safer and faster PVD.

## Conclusions

In contrast with previous techniques, the “lightning bolt” or “Kaminari Kagura technique enables a smooth and gentle dissection of the posterior hyaloid. The advantage of this technique is the possibility of having a standardized, repeatable, and easy method to separate the posterior hyaloid from the retina surface without the risk of creating holes, haemorrhages, or unwanted retinotomies.

### Supplementary Information

Below is the link to the electronic supplementary material.

Supplementary file1 (MOV 108348 kb)
